# Mr. Earnest's Case of Dropsy

**Published:** 1806-06

**Authors:** R. Earnest

**Affiliations:** House Surgeon to the Sheffield General Infirmary


### *To the Editors of the Medical and Physical Journal.*

Gentlemen,

It is a custom with with me whenever any thing occurs in practice appearing extraordinary, to minute it down at the time; the enclosed is an account of a very complicated case of dropsy, which I have selected from amongst several others of that species of disease; some remarks are also added upon the speedy specific effects of opium, the warm bath, &c. in Nephritis, which the patient was attacked with during her stay at this Infirmary. If you think the case merits a place in your useful Journal, you will much oblige me by inserting it in the next number.

I am, &c.

*April* 20, 1806. R. EARNEST.

House Surgeon to the Sheffield General Infirmary.

### CASE.

E. S. a woman forty-three years of age, married, and has had several children, and also several abortions; she was low in stature, and her habit leucophlegmatic. After labouring under hydropic complaints upwards of eight years, she was at length admitted into the Sheffield General Infirmary, in December 1803; the symptoms at that time were as follow, namely, The pulse was small, quick, and irregular; the skin yellow, hot, and dry; great thirst, the appetite diminished, the urine small in quantity, thick and high coloured; the bowels were costive; the right hypochondrium was more full and tense than the left, and to the touch the great lobe of the liver felt to be in a state considerably enlarged; respiration was greatly incommoded, and appeared that kind of dyspnœa which is commonly observed in hydrothorax; and I cannot hesitate a moment in saying, that that was the case here; there was also ascites, general anasarca, and great prostration of strength.

At this time, December 16, the patient was put upon the following medicines, viz. Calomelas, pulvis digitalis. pulv. scillæ. spiritus ætheris nitrosi. spiritus juniper comp. along with an aromatic bitter infusion; cremor tartar. which were varied, and omitted, according to circumstances and symptoms. A generous diet was conjoined. These remedies were persisted in until the 25th of January 1804,* when she was attacked violently with nephritis; and the symptoms that now took place were truly formidable, namely, constant and acutely painful dysuria, excruciating pain about the region of the kidnies, extending all around the abdomen; nausea, retchings, and frequent fits of syncope; cold colliquative sweats; the pulse quick, small, and trembling; the thirst was much increased, and the patient had a strong and frequent desire for hot spirituous cordials; she could not lie in a horizontal posture.

Although at this period the inflammatory symptoms ran alarmingly high, and seemingly to demand the use of the lancet, but after recapitulating the state of the patient with the symptoms first detailed, and the inference will

* In the course of forty-one days, namely, from December 16th to January 25th, the patient took many very active medicines, but the functions in general were become so blunted by long continued disease, as to resist the power of any medicine; consequently she appeared not all at benefitted by them.

be be that the patient was not a proper subject to bear the loss of blood; therefore in lieu, of phlebotomy, refrigerants, &c. the following plan was immediately administered, namely,

1. The warm bath to 98° of Fahrenheit's thermometer.
2. ℞. Mucilag. amyli. unc. iv. tinct. opii gutt. LX. M. fiat enema.
3. ℞. Aq. menth. piper. unc. j. tinct. lavend. c. gutt. xx. tinct. opij. gutt. xv ad xxx. M. fiat haustus.
4. ℞. Ol. Ricini.
5. ℞. Mistur. camphorat. unc. vii. spt. juniper. comp. unc. 1. confect. aromat. dr. j. M. fiat mistura.

The first immersion in the warm water, the use of the enema, and the first draught, procured almost immediate relief in every respect; and about three hours after these remedies had been administered, the patient was pretty free from pain. The pains, &c. returned several times, but in a less violent degree, and a repetition of the same remedies never failed presently to relieve the patient. At intervals, when she was pretty free from pain, a large table spoonful of the Ol. ricini was taken, and when she was faint and languid, two table spoonsful of the mixture was given. She passed a tolerably easy night, and in the course of the next day, January 26th, when she was passing water, she parted with a small oblong renal calculus (which I suppose had passed the ureter the preceding day) about the size of a barley-corn, whose surface was very rough, and had several sharp points about it. This being extricated, the dysuria was cured—as also were the pains of the stomach and abdomen, &c.

All this accidental disturbance to the system made no change apparently in the hydropic complaints, but the patient's strength was much diminished, and her spirits a good deal exhausted by the attack.

I must say, that I never before witnessed a patient who suffered more during each paroxysm, the pains were so truly torturing; nor do I ever remember, at any time, having experienced so much real pleasure as in this instance, by the speedy and specific effects of the remedies employed; and I trust, that in future, when similar cases again occur, that the same remedies will be crowned with success equally satisfactory. They both apparently took off the violent spasms and promoted a gentle perspiration; the opium allayed the violent pains more deeply seated, and at the same time greatly facilitated the passage of the calculous concretion from the kidney; and the ol. ricini. proved proved a valuable auxiliary as a demulcent, and by gently evacuating the bowels.

I conclude this history by adding, that the patient was kept at the Infirmary from December 16th, 1803, until February the 24th, 1804. Mercury, the various evacuants and diuretics, the milder tonics and frictions, were tried in vain; and, I am sorry to say, that the patient was discharged not relieved. Sometime afterwards I was informed that she died, six weeks subsequent to leaving the Infirmary.

